# Why an Integrated Approach to Tick-Borne Pathogens (Bacterial, Viral, and Parasitic) Is Important in the Diagnosis of Clinical Cases

**DOI:** 10.3390/tropicalmed9110272

**Published:** 2024-11-11

**Authors:** Raúl Contreras-Ferro, Jorge Martín Trueba, Patricia Sánchez-Mora, Raquel Escudero, María Paz Sánchez-Seco, Estrella Montero, Anabel Negredo, Luis Miguel González, Alejandro Dashti, María Teresa Llorente, Judit Gil-Zamorano, Ana Vázquez, Isabel Jado, David González-Barrio

**Affiliations:** 1 Reference and Research Laboratory on Special Pathogens, National Center for Microbiology (CNM), Carlos III Health Institute (ISCIII), 28220 Madrid, Spain; raconfer01@gmail.com (R.C.-F.); jorge.martin@isciii.es (J.M.T.); rescude@isciii.es (R.E.); mtllorente@isciii.es (M.T.L.); judit.gil@isciii.es (J.G.-Z.); ijado@isciii.es (I.J.); 2 Arboviruses and Imported Viral Diseases Laboratory, National Center for Microbiology (CNM), Carlos III Health Institute (ISCIII), 28220 Madrid, Spain; patricia.sanchez@isciii.es (P.S.-M.); paz.sanchez@isciii.es (M.P.S.-S.); anabelnegredo@isciii.es (A.N.); a.vazquez@isciii.es (A.V.); 3 CIBER Enfermedades Infecciosas (CIBERINFEC), 28029 Madrid, Spain; 4 Parasitology Reference and Research Laboratory, National Center for Microbiology (CNM), Carlos III Health Institute (ISCIII), 28220 Madrid, Spain; estrella.montero@isciii.es (E.M.); lmgonzal@isciii.es (L.M.G.); dashti.alejandro@gmail.com (A.D.); 5 CIBER Epidemiología y Salud Pública (CIBERESP), 28029 Madrid, Spain

**Keywords:** tick-borne diseases, syndromic diagnostics, emerging pathogens, wildlife, public health, zoonoses

## Abstract

Tick-borne diseases have emerged as a major global public health problem in recent decades. The increasing incidence and geographical dissemination of these diseases requires the implementation of robust surveillance systems to monitor their prevalence, distribution, and public health impact. It is therefore not unexpected that tick-borne pathogens coexist in the same vectors, but the interactions of these agents between vectors and vertebrate hosts, including humans, remain poorly understood. The impact of infection in humans extends to the diagnostic challenges that arise when the same symptomatology can be associated with any tick-borne pathogen, and therapeutic recommendations only focus on the major or best-known tick-borne diseases, ignoring other lesser-known or less prevalent infections. Both surveillance systems and the holistic diagnosis of tick-borne pathogens are necessary tools to address the emergence of vector-borne diseases. In this study, we will focus on the main tick-borne viral, bacterial, and parasitic diseases in Spain to reflect the need to establish syndromic diagnostics in samples from patients with a history of tick bites and symptomatology compatible with them. On the other hand, and highlighting this need, innovations in molecular techniques, syndromic surveillance, and surveillance programs for ticks and tick-borne pathogens with public health implications are expected to be developed.

## 1. Introduction

The number of zoonotic pathogens that can infect humans by different routes of transmission (from animal reservoirs, through food, or by vectors) has been increasing in recent decades [1]. One of the main causes of this increase are anthropogenic activities including land use change, increasing urbanization, the intensification of agricultural systems, and deforestation, among others [2]. The transmission of these pathogens to humans has been strongly favored by the development of new connections, which can be classified as urban–peri-urban–rural, human–animal–ecosystem, or, from a broader perspective, as an urban–wild relationship [3]. These are environments in which the cycles of pathogen spread between species take place. In this situation, changes in ecosystems force human and animal populations to inhabit areas that are unfavorable for their development or where they may face infectious organisms without having had a previous relationship with them and, therefore, lack immunological barriers or defenses, and which have been fundamental for the occurrence of some vector-borne infections [3]. These events have led to the appearance or emergence of new pathogens (generally of zoonotic origin) and the re-emergence of supposedly controlled infectious agents transmitted by reservoirs (wildlife) or vectors (mosquitoes, fleas, and ticks) [4,5].

Tick-borne diseases (TBDs) have increased in recent years. Until barely two decades ago, the spectrum of tick-borne diseases was limited to a few infectious processes confined to certain geographical areas [6]. Currently, clinical–epidemiological observations and, above all, the incorporation of molecular biology techniques have allowed for the description of new infectious processes and descriptions of the implications in the human pathology of different pathogens, both in isolation and in co-infection [7]. Tick-borne co-infections are the result of infections with genetically different pathogens, which may be closely related, such as variants within the same species through to diversely different pathogens, such as parasites and bacteria or viruses. In humans, and after a tick bite, a variety of non-specific symptoms may appear, such as headaches, fever, sweating, dizziness, nausea, vomiting, and muscle pain. These are very early signs that can be produced by any tick-borne pathogen [8]. An example may be Lyme disease, which remains the most common tick-borne disease, and co-infection, most commonly with babesiosis, occur in up to one third of *Borrelia* infections [9]. Several factors raise suspicion of co-infection, such as laboratory abnormalities and prolonged duration of symptoms despite adequate treatment. Clinicians should maintain a high level of suspicion of co-infection, as untreated disease can lead to prolonged and sometimes fatal infection [9,10]. The need for effective and holistic early detection of these pathogens in public health would improve not only the entire National Health System, saving costs that would increase if only focused on individual diagnosis, and reducing the waiting time for diagnosis, but also reduce the application of adequate treatment and improve the patient’s prognosis.

This paper has reviewed tick-borne diseases in Spain and has made an exhaustive review of the requests received over the last ten years by the National Centre for Microbiology. It also recommends the unification and implementation of holistic diagnostics using new molecular technologies that benefit the National Health System, clinicians, and, most importantly, patients with these diseases.

### 1.1. Current Situation of Tick-Borne Diseases in Spain

The strategic situation of the Iberian Peninsula as a bridge between two continents and the common space it forms with other Mediterranean countries is responsible for a unique biodiversity which also affects ticks. Thus, in a relatively small space, more tick species coexist than in the rest of Central Europe and, therefore, also a greater variety of TBDs (Table 1) [11,12,13,14,15,16,17,18,19,20,21,22]. Tick-borne pathogens (TBPs) are a major public health concern, and co-infection with multiple pathogens occurs frequently, posing a serious challenge for proper diagnosis and treatment [9,10,23,24,25,26]. It is important to note that the diagnosis of tick-borne diseases is complex and requires a comprehensive approach. As the incidence of these diseases increases, it becomes more important for health care professionals to distinguish between the different clinical presentations of these processes. The clinical presentation, mostly non-specific at the onset of infection with most of these diseases, and the possibility of co-infection by several pathogens (Table 1), as well as the detection of new pathogens, does not favor the orientation of a concrete and confirmatory diagnosis, which is necessary to make a differential diagnosis that, in most cases, is not determined. Due to the above, as well as to the seriousness of some of the clinical pictures and the sequelae and handicaps that these diseases can cause, the attention that should be paid to these pathogens and ticks is justified. In case of suspicion of tick-borne disease, it is necessary to group this in the diagnosis the main TBP, including bacterial, viral, and parasitic agents. To be able to correctly diagnose a TBD, one needs to take into account potential co-infection(s). This is necessary when selecting the correct treatment and because of the possibility that a co-infection may aggravate the illness [27].

Some of the most common TBDs in Spain are listed below, detailing their main characteristics. Since ticks can harbor bacteria, parasites, and viruses, and these diseases are divided into three different groups according to the causative agent.

### 1.2. Tick-Borne Diseases Caused by Bacteria

#### 1.2.1. Lyme Borreliosis

Lyme borreliosis is the best-known tick-borne disease, and that may be because it is the most prevalent tick-borne disease in the Palearctic region of the Northern Hemisphere [28]). Caused by the genospecies of *Borrelia burgdorferi* sensu lato complex, in Europe, *Borrelia afzelii* and *Borrelia garinii* are mostly associated with human disease. There are 85.000 new cases estimated to occur every year throughout Europe, with cases peaking in summer. This rate is parallel to the densities of *Ixodes ricinus* ticks present in these regions. However, the increased distribution and abundance of ticks due to climate change and increased awareness is causing Lyme disease cases to increase [29]. Humans are considered accidental hosts when their activities interfere with the spirochete cycle. These ticks have a two–four-year life cycle with four stages: egg, larva, nymph, and adult. Larvae acquire the bacteria through the feeding of an infected host. At the nymph stage they are able to infect other healthy hosts [29]. Infection begins with the tick bite, through which the spirochetes found in the tick’s gastrointestinal system (approximately 20 spirochetes per bite at peak infectivity) can be transmitted. Between 7 and 14 days after the bite, they begin to replicate along the skin. They are able to spread throughout the tissue faster than macrophages, so they are able to escape from them [29]. The most recognizable symptom of infection is erythema migrans (EM). The speed at which the erythema moves is related to the speed of the spirochetes (20 cm per day). Parallel to migration through the skin, spirochetes also enter and infect the bloodstream. Lyme borreliosis is known to affect a multitude of organs, but the mechanisms by which spirochetes invade these organs are not well understood. Apart from the skin, the heart, nervous system, and joints are the most commonly affected sites. However, the rates of involvement present a major challenge, as different sources provide different information. The Centers for Disease Control and Prevention (CDC), for example, estimates that 1% of patients have cardiac involvement, which is much lower than in animal models. With this in mind, Lyme borreliosis has been divided into three stages: (i) Erythema migrans (lymphocytoma), (ii) Neurological or cardiac involvement, and (iii) Arthritis. It is important to know that only a small group of untreated patients show all of the possible manifestations.

(i) On stage one, several EM-associated systemic illnesses may occur, such as arthralgia, malaise, fatigue, headache, and low-grade fevers and chills. Regional lymphadenopathy could also occur. The agent is very important to the development of the disease. For example, patients with *B. garinii* had shorter incubation periods, a faster evolution of EM, and more symptomatic lesions. Only in Europe are there two additional dermatologic disorders: Borrelial lymphocytoma and acrodermatitis chronica atropicans. The first can appear several weeks after EM, while the second is extremely late, typically years after [29].

(ii) The neurological symptoms are varied. At first, they usually affect the cranial and peripheral nerves associated with lymphocytic meningitis. Classical Bannwarth syndrome (BS) may appear, and it is the most common neurological affection that Lyme borreliosis causes in Europe. Scientists have hypothesized that neurotropic strains are more common in Europe. Other neurological affections that are completely linked to Lyme disease are encephalomyelitis, encephalopathy, and axonal polyneuropathy [29]. Cardiac involvement does not occur if the disease is treated early, and so far occurs in only 1% of patients. The most common manifestation is an atrioventricular block proximal to the bundle of His. Other affectations that have been noted are atrial fibrillation, sick sinus syndrome, and diffuse myocardial involvement. Sudden death is extremely rare but possible [29].

(iii) Arthritis is the most common and serious rheumatologic consequence of *B. burgdorferi* complex dissemination through the body. Incubation in the joints may vary from days to years. In some patients, pain appears weeks or even months before arthritis. It is characterized by episodes of inflammation with swelling and large effusions but little pain. The knee is the most commonly affected, but other joints may suffer too. In Europe, the number of individuals that develop arthritis is less than 10%, in contrast with the US, with 30%. Although arthritis usually resolves after antibiotic treatment, some patients have persistent proliferative synovitis that causes damage, intense inflammation, and fibrosis of the joint [29].

#### 1.2.2. Mediterranean Spotted Fever and Mediterranean Spotted Fever-like

Mediterranean spotted fever (MSF) is a zoonotic disease endemic to the Mediterranean area. Its etiological agent is well known: the bacterium *Rickettsia conorii*. The vector that transmits MSF to humans is the brown dog tick, *Rhipicephalus sanguineus*. The natural cycle of *Rickettsia conorii* involves the tick having a central role. The ticks themselves can be infected in three different ways: (1) feeding from an infected animal, (2) trans-ovarially, and (3) transstadially. Despite all these processes of infection, only 15% of ticks have been observed to have the infection [30]. The prevalence of MSF exhibits temporal variability within endemic regions, displaying fluctuations with peaks and troughs over recent decades. Epidemiological data also suggest a seasonal pattern in MSF endemicity, with a higher prevalence during the summer months. The influence of elevated temperatures on tick behavior intensifies the quest for hosts throughout all developmental stages, and subsequently heightens the risk of human exposure to the disease [30].

The classic triad of MSF symptoms includes fever, maculopapular rash, and an inoculation eschar at the tick bite site. Fever is nearly universal, typically appearing after an incubation period of around six days, though it can vary from one to sixteen days. Early disease symptoms encompass headache, arthralgia and myalgia, local lymphadenopathy, hepatomegaly, splenomegaly, and gastrointestinal manifestations. Most patients develop a sparse macular rash that evolves into a maculopapular pattern, typically involving the palms and soles while sparing the face. The rash usually emerges two to three days after the onset of fever, but its onset may be delayed until the fifth day. In rare instances (1–4%), the rash may be absent, and approximately 10% of patients may exhibit a petechial rash with occasional vesicular exanthema [30]. Typically, MSF follows a self-limited course lasting 12 to 20 days, although hospitalization is not uncommon. With treatment, symptoms begin to subside within 48 h, and complete recovery is usually achieved within 10 days. There is no chronic form of the disease. While MSF generally has a mild course, complications may arise in 1% to 20% of patients, with a case fatality rate of 0% to 3% in most series [30]. Life-threatening complications of MSF include cardiac symptoms (coronary ectasia and atrial fibrillation), neurological manifestations (cerebral infarct, meningoencephalitis, and sensorineural hearing loss), renal failure, intraocular inflammation, pancreatitis, and other multi-organ complications [30]. Indirect immunofluorescence antibody is the test of choice. For a definitive diagnosis, two positive serum samples in a span of four weeks are needed. For early diagnosis, molecular techniques are the first choice. PCR is usually used for this early diagnosis. Both tissue and whole blood can be used. On the other hand, other *Rickettsiae* species such as *R. monacensis*, *R. massiliae* and *R. aeschlimannii*, and *R. sibirica mongolitimonae* are also known to cause diseases similar to Mediterranean spotted fever and are known as Mediterranean spotted fever-like and Lymphangitis-Associated Rickettsiosis (LAR), respectively [14].

#### 1.2.3. Dermacentor-Borne Necrosis Erythema Lymphadenopathy (DEBONEL)-Tick-Borne Lymphadenopathy (TIBOLA)-Scalp-Eschar-and-Neck-Lymphadenopathy-After-Tick-Bite (SENLAT)

This vector-borne disease has several designations, mainly DEBONEL (*Dermacentor*-borne necrosis erythema lymphadenopathy) and TIBOLA (tick-borne lymphadenopathy), and can also be named SENLAT (scalp-eschar-and-neck-lymphadenopathy-after-tick-bite). *Dermacentor marginatus* serves as the primary vector for DEBONEL/TIBOLA, although *D. reticulatus* has also been implicated [31]. In terms of etiological agents, *Rickettsia slovaca* was first detected via polymerase chain reaction (PCR) in 1997 of a French patient who exhibited a scalp eschar and lymphadenopathy after being bitten by a tick in the Pyrenees Mountains (France). Six years later, in 2003, the culture and isolation of *R. slovaca* from another French patient were reported, confirming *R. slovaca* as a human pathogen and an etiological agent [31]. Following a bite from a *Dermacentor* spp. tick, a significant proportion of patients develop an inoculation eschar, characterized by a point of necrosis at the site of the tick bite. This eschar is typically surrounded by erythema and accompanied by regional enlarged and painful lymphadenopathies [31].

According to Silva-Pinto et al., (2014) [32] almost 95% of the tick bites from the vector affected the head, particularly the scalp, with 316 scalp bites compared to 17 non-scalp bites (on the trunk, upper limb, and lower limb). The majority (88.9%) of these tick bites resulted in an eschar, sometimes accompanied by inflammatory signs such as erythema. The incubation period varied widely, but was typically around 1–2 weeks. Among the reported symptoms, fever was present in 139 cases (26%), headache in 84 cases (16%), and persistent asthenia in 41 cases (7%). Rash (13 cases, 2%), myalgia (24 cases, 4%), and vertigo (3 cases, 0.6%) were less common. Scarring alopecia was the most common sequela, reported in 100 cases (19%). Lymphadenopathy occurred in most cases, typically being painful and localized in the cervical or occipital areas

#### 1.2.4. Human Granulocytic Anaplasmosis

Human Granulocytic Anaplasmosis (HGA) is an emerging tick-borne disease. Its etiological agent is the bacteria *Anaplasma phagocytophilum*. Biological vectors include ixodid ticks that include different genera. Though *Anaplasma* usually shows host specificity, to some degree, it has been found that *A. phagocytophilum* can be found in a wide range of animal species, including humans [33]. The life cycle of *Anaplasma* is not well studied, and more information is needed to fully understand it. It is known that infected ticks can inoculate *Anaplasma* spp. when feeding on blood. Depending on the specific *Anaplasma* species, the target cell may be leukocytes, erythrocytes, or platelets. Intranuclear inclusion bodies form inside these cells and spread the bacteraemia in the bloodstream. When an uninfected tick feeds on the blood of an infected animal, the bacteria settle in the intestinal lumen, where they begin to replicate and migrate to the salivary glands [33]. The variability in the severity of HGA is notable, with some individuals exhibiting no symptoms, while others experience a non-specific febrile illness. Severe disease is rare, affecting only a minority of cases. The overall case fatality rate is approximately 0.6%. Common symptoms include fever, malaise, headache, and myalgia. Gastrointestinal symptoms are also prevalent, with patients reporting diarrhea, nausea, vomiting, anorexia, or abdominal pain. Although European cases may tend to be milder compared to those in the United States, they still exhibit a substantial hospitalization rate, reaching nearly 63%. Rash is a less common presentation of HGA, unlike in other tick-borne diseases such as Lyme disease. In addition to typical symptoms, uncommon presentations of HGA include myocarditis, seizures, short-term memory impairment, orchitis, glomerulonephritis, myositis with severe rhabdomyolysis, peripheral neuropathy at the tick bite site, cerebral infarct, and hemophagocytic lymphohistiocytosis (HLH) [33]. The most effective method for diagnosing HGA is the PCR of whole blood. An alternative diagnostic approach is the microscopic examination of Giemsa-stained peripheral blood smears, which can reveal morulae (clusters resembling blackberries) within polymorphonuclear leukocytes (PMN). In addition, serology plays an important role in diagnosis, where *Anaplasma*-specific IgM and/or IgG antibodies are detected by immunofluorescence. However, it is important to note that antibodies may not be uniformly present during the acute phase of infection. In such cases, the diagnosis is confirmed by demonstrating a four-fold increase in IgG levels, with at least four weeks between tests. It has been occasionally described in the north of the Iberian Peninsula; however, high infection rates in mammals suggest that it is an under-diagnosed disease in Spain [34].

### 1.3. Other Possible Tick-Borne Bacterial Diseases

#### 1.3.1. Tularemia

Tularemia is a vector-borne disease, the causative agent of which is *Francisella tularensis*. There are several subspecies that make up the genera. In Europe, the leading cause of Tularemia is *F. tularensis subsp. holarctic* [15]. It is primarily a zoonotic disease that occurs through contact with infected animals (direct or indirect). There are several reservoirs, such as rodents, voles, and water voles, which are among the major sources of infection because they excrete the bacteria and they contaminate the water. This is very important due to the fact that infection with *F. tularensis* happens mainly through infected water [15]. However, and even though it is well known as a tick-borne disease, it is usually not immediately recognized after a tick bite [35]. In Europe, important tick vectors are *D. nuttalli*, *D. marginatus*, *I. ricinus*, *D. reticulatus*, and *Haemaphysalus concinna*. Tularemia demonstrates a seasonal trend in countries characterized by relatively cold climates, with the majority of cases reported between July and November. This aligns with an increased probability of exposure to the organism or vectors during outdoor recreational activities [15]. The incubation period ranges between 3 and 14 days, though the usual period is normally 3–5 days. The general symptoms are fever, malaise, chills, and headache. However, it manifests in six classical forms in humans, each determined by the site and route of infection [36]. Here, we will focus on the symptomatology due to vector bites. Thus, it may occur in the following forms. (i) Ulcero-glandular form: infection via direct contact with an infected animal or vector bite leads to this form, featuring symptoms such as skin lesions and lymphadenopathy. (ii) Glandular form: similar to the ulcero-glandular form in transmission, this form is distinguished by regional lymphadenopathy without detectable skin lesions.

The diagnosis of tularemia primarily relies on positive serology combined with clinical and epidemiological contexts. PCR-based methods are useful when tissue samples are available, allowing for the confirmation of *F. tularensis* presence through the amplification of target nucleic acid sequences [15].

#### 1.3.2. Q Fever

Q fever is a globally prevalent zoonotic disease. The etiological agent, *Coxiella burnetii*, exhibits a broad spectrum of hosts and demonstrates remarkable resilience to adverse environmental conditions, including aridity, high temperatures, and disinfectants, thereby posing a prolonged risk of infection [37]. The primary mode of dissemination is through airborne transmission, facilitated by contaminated air or dust. Despite its widespread nature, the major reservoir and potential threat to humans are attributed to sheep and goats [38]; however, a significant number of human Q fever cases show neither direct nor indirect exposure to livestock as a risk factor, but rather exposure to wildlife [39]. The high host abundance can be observed in ticks, as *C. burnetii* has been found in ticks of several genera, such as *Dermacentor*, *Haemaphysalis*, *Hyalomma* (the most abundant), *Ixodes*, and *Rhipicephalus* [40]. Historically, it has been considered a vector-borne disease, but the role of ticks in transmission has not fully been elucidated yet. The excretion of *C. burnetii* in tick feces and saliva is well documented, but the role of these findings or the epidemiological context is discussed controversially [41]. Human infection manifests in both acute and chronic states. The fever associated with Q fever typically persists for 9–14 days, but approximately 60% of infected individuals remain asymptomatic. Only 5% may progress to endocarditis. Common Q fever manifestations include flu-like symptoms, anorexia, upper respiratory tract issues, persistent cough, chest pains, confusion, and occasionally nausea and diarrhea. Notably, Q fever fatigue syndrome, affecting 20% of patients, has been described as a debilitating chronic condition with economic consequences, but it is under discussion [42]. In cases of chronic infection, the occurrence of endocarditis, as previously mentioned, is observed, along with additional complications such as pericarditis, myocarditis, osteomyelitis, nephritis, hemolytic anemia, severe headaches, thyroiditis, and hepatitis, among other rarer symptoms. During pregnancy, infections often progress asymptomatically. However, noteworthy obstetric complications may arise, including placentitis, spontaneous abortion, impaired fetal growth, stillbirth, premature delivery, and the birth of fragile offspring. Infections contracted during pregnancy can also contribute to miscarriages in subsequent gestations [43,44,45,46]. Diagnosing Q fever is a complex task, given the challenges associated with relying on clinical signs, symptoms, or post-mortem examinations due to the non-specific nature of the disease presentation and the absence of distinctive symptoms and lesions. Therefore, the precise identification of *C. burnetii* infection hinges on laboratory evidence.

### 1.4. Tick-Borne Diseases Caused by Parasites

#### Babesiosis

Babesiosis is a zoonotic illness that affects the USA, Europe, and Asia, with *Babesia divergens* being a prominent species found primarily in Europe, with a percentage of ticks infected with *Babesia* spp. ranging from 0.78% to 51.78% [18]. *Babesia divergens* is primarily transmitted by *Ixodes ricinus* ticks, in Europe, with cattle serving as the primary reservoir host. Interestingly, *I. ricinus* also acts as a reservoir though transovarial transmission, as *B. divergens* parasites are transmitted from gravid female ticks to their eggs, infecting subsequent life stages without the need for a mammalian host. Transstadial transmission, which occurs between different life stages, is also important, with the nymph stage being the primary vector of infection [47]. *Ixodes ricinus* is also the vector for the autochthonous zoonotic species *Babesia microti* and *Babesia venatorum* in Europe. Both species cause human babesiosis, mainly in immunocompromised patients, which possibly suggests a milder course of infection than *B. divergens* [48].

Clinical manifestations of babesiosis vary widely, ranging from asymptomatic infection to life-threatening disease. After an incubation period of 1–3 weeks, symptoms generally have a rapid progression. Patients often experience malaise and fatigue, followed by a combination of symptoms, including intermittent fever of up to 40 °C, chills, sweats, anorexia, headache, and myalgia. Less common symptoms may include arthralgia, emotional instability, depression, hyperesthesia, neck stiffness, sore throat, nausea, abdominal pain, vomiting, conjunctival injection, photophobia, weight loss, shortness of breath, and non-productive cough. Physical examination findings occasionally include mild to moderate splenomegaly, hepatomegaly, and pallor and/or jaundice. Severe babesiosis is more common in asplenic and immunocompromised patients, and they are at higher risk of complications such as severe anemia, acute respiratory distress syndrome, disseminated intravascular coagulation, congestive heart failure, renal failure, hemophagocytic lymphohistiocytosis, and coma resulting in death. However, publications of exceptional cases of severe babesiosis in young healthy patients in France and Spain have challenged this “classic description of babesiosis in Europe” [49,50,51]. The mortality rate is as high as 42% in severe cases in asplenic and immunocompromised individuals who are infected with *B. divergens*.

In Europe, misdiagnosis and a lack of awareness of babesiosis have occasionally led to delayed diagnosis [48]. The diagnosis of babesiosis can be confirmed by the microscopic detection of parasites within red blood cells on Giemsa-stained or Wright-stained thin blood smears. PCR is more sensitive than a blood smear for detecting *Babesia* [52]. Unfortunately, no standardized diagnosis tests are available in Europe so far [48].

Despite the high abundance of *I. ricinus* ticks and a seroprevalence rate of 39.2% in the local population in Spain, the number of clinically diagnosed babesiosis cases is low [10]. During the period spanning January 1997 to December 2019, Spain recorded a total of 29 cases. Although the distribution of cases was variable, there appeared to be a stabilization at 0.21 cases per 10,000,000 person-years in the final two years of the study [18]. Consequently, babesiosis remains a rare infection in Spain, but there is increasing evidence for persons with *Babesia*-positive antibodies, asymptomatic patients, and patients with fulminant and fatal babesiosis [10,51,53,54,55].

### 1.5. Tick-Borne Diseases Caused by Viruses

#### Crimean-Congo Hemorrhagic Fever

The etiological agent of Crimean-Congo hemorrhagic fever (CCHF) is a virus of the *Nairoviridae* family, *Nairovirus* genus, and is the most lethal tick-borne hemorrhagic disease. Ticks are the main vectors responsible for the transmission of Crimean-Congo hemorrhagic fever virus (CCHFV), with particular emphasis placed on ticks of the genus *Hyalomma* [56]. These ticks often parasitize several vertebrate species, both wild and domestic mammals. Consequently, animals such as cattle, goats, and sheep serve as a reservoir for viral amplification, often showing only transient viremia. The virus can persist within ticks throughout their lives through mechanisms such as trans-stage transmission [57]. Humans can contract CCHF through the bite of an infected tick or through direct contact with the mucous membranes or body fluids of infected individuals or animals. Although CCHF represents the most severe consequence of CCHFV infection, seroepidemiological analyses have prompted a reassessment of asymptomatic CCHV infections in humans, leading to a broader understanding of the clinical spectrum of the disease [57].

The incubation period varies depending on the route of exposure, lasting 1 to 9 days when acquired by tick bite and 5 to 13 days if acquired by contact with contaminated tissues or blood. This may be followed by the first symptoms, which may include a significant increase in body temperature, chills, photophobia, myalgia, nausea, and severe headache, and a short hemorrhagic phase (2–3 days) characterized by various signs such as petechiae, ecchymosis, and potentially dramatic hemorrhages in the gastrointestinal, urinary, cerebral, and respiratory tracts. This phase is associated with negative prognostic indicators [58].

Diagnosis of CCHF usually begins with a thorough collection of information about the patient, including recent travel history and a medical examination. Currently, laboratory methods for the diagnosis of CCHF include real-time polymerase chain reaction (RT-PCR) to detect viral RNA in patient samples and virus isolation, although less frequently used due to its complexity and time-consuming nature, and fundamentally because laboratories of the highest biosafety level (i.e., BSL4) are required, and can provide definitive evidence of CCHF infection [57].

Early recognition of symptoms, prompt medical assessment, and appropriate supportive measures are essential to improve patient outcomes and reduce the mortality associated with CCHF, as well as the need for rapid diagnosis to implement effective isolation and public health measures for the containment of infection [57].

In Spain, where most CCHF patients have been diagnosed since 2016, the fatality rate of CCHF was as high as 30%. Of note, those 3/10 patients who died showed the highest Bakir-scale scores (>7) on admission. Previous studies have shown that in this viral infection transmitted by ticks, regional differences in mortality rates may be related to factors including the availability of advanced medical care facilities, faster diagnosis because of a better surveillance system that enables the early detection of cases with mild to moderate clinical findings, the routes of acquisition of the infection, and the genotype of the virus [17].

## 2. Retrospective Study Conducted on the Laboratory Diagnosis Database of the National Centre for Microbiology, Carlos III Institute of Health (CNM-ISCIII), from 2014 Through to 2023

**Figure 1:**
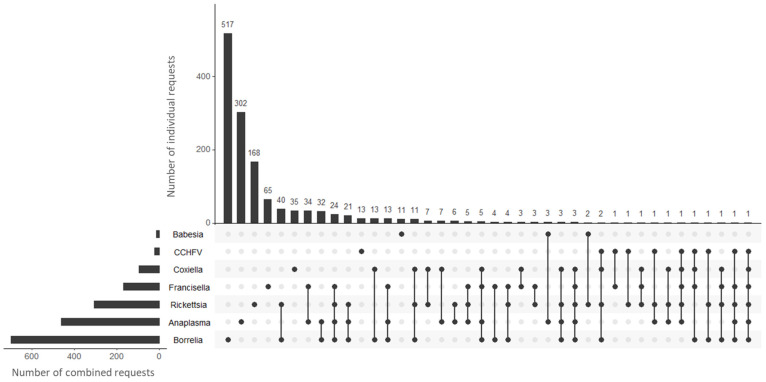
Individual and combined request numbers received by the National Center for Microbiology from 2014 through to 2023 for the different TBPs.

Retrospective analyses may provide an opportunity to observe patterns in requests related to infection with tick-borne pathogens. In this context, and in order to restrict requests, only requests where both the history and observations described by the clinicians indicated a confirmation of arthropod/tick bites in the patients (no other information was extracted from the database to protect the anonymity of the patients). This search counted both individual and combined requests for the detection and exposure of different TBPs, including bacteria such as *Borrelia* spp., *Rickettsia* spp., *Anaplasma* spp., *Coxiella burnetii*, and *Francisella tularensis*, and parasites such as *Babesia* spp. and Crimean-Congo hemorrhagic fever virus. Overall, the number of orders for testing for the presence of nucleic acids and/or antibodies to TBPs in patients who had been bitten by an arthropod/tick and with present symptoms amounted to 1368, of which 81.2% (1111/1368) were requests for a single pathogen, with *Borrelia* being the highest request, with more than 500 request in the last 10 years, followed by *Anaplasma* and *Rickettsia,* with 302 and 168 petitions, respectively. Figure 1 shows the individual and combined request values of the different pathogens. Requests with two or more pathogens in combination accounted for 18.8% (257/1368) of the requests. Among these, requests of two, three, four, and five pathogens account for the 61.1%, 22.6%, 14.0%, 1.9%, and 0.4%, respectively, being the combinations of requests for *Borrelia-Rickettsia* (n = 40), *Anaplasma-Francisella* (n = 34), and *Borrelia-Anaplasma* (n = 32), the most frequent.

## 3. An Overview of the Clinical Signs and Symptoms of Tick-Borne Diseases in Spain: The Challenge of Multiple and/or Syndromic Diagnosis

**Figure 2:**
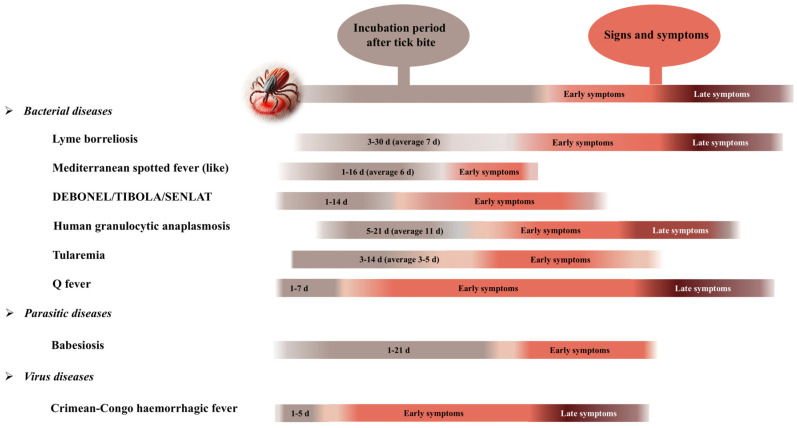
Timeline of the different tick-borne diseases.

With the above framework of diseases, pathogens, and symptomatology (Table 1, Figure 2), an integrated diagnosis by molecular techniques seems necessary, at least in the first days/weeks post-infection, where many of these tick-borne diseases present a very similar clinical picture (Figure 2).

**Figure 3:**
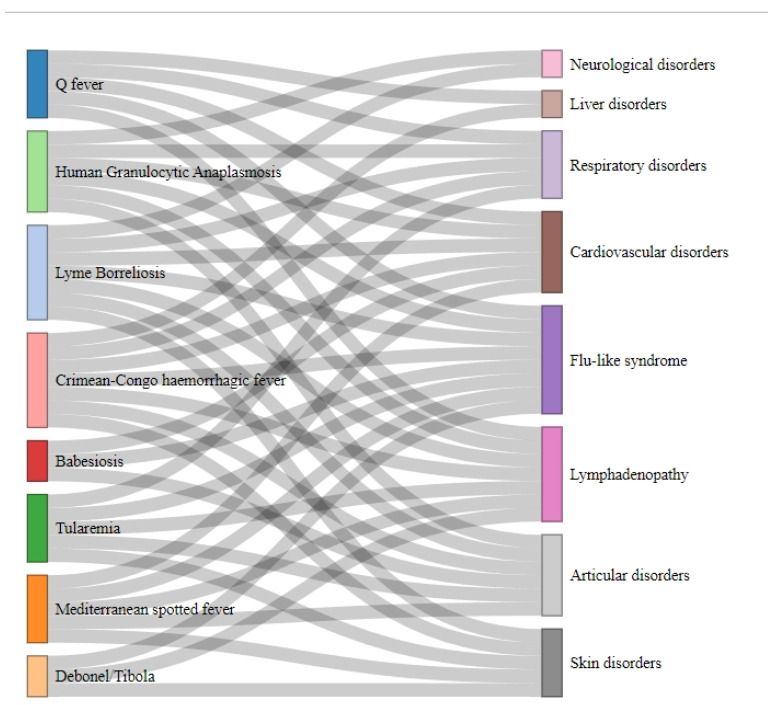
Sankey diagram showing the overlapping symptomatology in the different tick-borne diseases.

In this respect, we observe that many of the tick-borne diseases present a very similar symptomatology, starting with a flu-like syndrome, followed by lymphadenopathies and muscular and articular problems (Figure 2 and Figure 3). Diagnosis can be difficult if we only focus on one pathogen, overlooking all other TBPs that cause similar symptomatology, as we can see in Figure 2, where the initial symptomatologies between the different tick-borne pathologies are very similar, overlapping between diseases in their acute phase.

The traditional methods for detecting TBPs were culture, microscopy, and serological tests [59]. Although these tests are commonly used today, they have inevitable limitations, such as serological cross-reactivity between closely related organisms and the lack of culturability of some fastidious organisms or biosafety requirements, which restricts their usefulness in detecting emerging pathogens [59].

The development of molecular diagnostic tools, such as PCR and nucleic acid sequencing, has had a significant impact on our understanding of TBPs and their role in human disease [60]. Real-time PCR and new molecular techniques provide a rapid and highly sensitive method for the detection of TBPs, and can be used as a tool for the discovery of new pathogens [60,61]. The quality, diversity, and availability of diagnostic technologies have improved significantly in recent decades. However, their relatively recent characterization, limited resources, and ecological complexity have made diagnosis and surveillance a constant challenge [60].

One of the most obvious and well-known cases for implementing syndromic diagnosis is co-infection by *B. burgdorferi* s.l. and *Babesia* spp. [10]). Lyme borreliosis is one of the best-characterized TBDs and the subject of much public health attention. However, due to its diverse clinical presentation, it can easily escape recognition in the absence of its characteristic erythematous migratory rash or be confused with any other infectious process. On the other hand, infections between Lyme disease and babesiosis may occur in two-thirds of the cases of Lyme disease diagnosed in the USA [9]. Several factors suggest co-infection, including laboratory abnormalities and the prolonged duration of symptoms despite adequate treatment. In patients who are receiving adequate treatment for Lyme borreliosis but remain febrile for more than 24 h, it is recommended that co-infection with other tick-borne diseases be investigated. Co-infection screening is also recommended in patients with unexplained thrombocytopenia and/or anemia. Clinicians should have a high level of suspicion for co-infection, as untreated disease can lead to long-term and sometimes life-threatening sequelae [10]. More than two decades ago, in an article on co-infection rates with tick-borne diseases [62], 39% of patients were co-infected with more than one organism. The most common co-infection was Lyme disease with babesiosis (81% of co-infections), and only 5% of patients had three infections (Lyme, anaplasmosis, babesiosis). This may suggest that this percentage may currently be higher due to the increased distribution of ticks and the pathogens they carry. On the other hand, there may be cases in which the symptomatology may lead us to believe that it is clearly caused by a pathogen, such as the recent case in which the presence of *Rickettsia raoultii* has been described in a patient with erythema migrans, more closely related to *Borrelia* spp. infection [63]. Another case is described by Negredo et al., in 2021 [64], of a person (32-year-old previously healthy woman) who recovered from a severe illness in May 2013, described as ‘caused by a tick bite’, and whose etiology was unknown. The patient’s occupation did not expose her to animals. Her medical history was reviewed, and it was noted that three days after being bitten by a tick during a walk in the mountains, she requested medical attention after experiencing fever and chills. On the next day, the patient’s general condition deteriorated (arthromyalgia, nausea, vomiting, and diarrhea), and she was admitted to a local hospital. Physical examination revealed erythema and a necrotic lesion on the patient’s back in the area of the tick bite. Despite treatment, septic shock developed, and supportive treatment was started in the intensive care unit. After 10 days of hospitalization, the patient recovered and was discharged. The final laboratory diagnostic tests ruled out infection with the most common tick-borne diseases (i.e., *Rickettsia* spp., *B. burgdorferi* s.l., *Anaplasma* spp., and *Ehrlichia* spp.), and other suspected etiologies (i.e., cytomegalovirus, *Coxiella* spp., hepatitis C virus, hepatitis B virus, HIV). Eight years later, and thanks to research on Crimean-Congo viral hemorrhagic disease, this case could be confirmed as the first autochthonous case of Crimean-Congo viral hemorrhagic disease [64]. These remarkable cases may only be the tip of the iceberg of the presence of TBP in the Spanish population, and further research is needed in syndromic diagnosis covering all possible TBPs, including both known and potentially unknown pathogens.

For patients, these inaccurate, erroneous, or late diagnoses pose a risk of developing more severe and chronic forms of disease. Indeed, many unexplained syndromes associated with tick bites have led to considerable discrepancies between infectious disease institutions and patient associations. For public health authorities, these limitations result in fragmented data sets, subject to significant spatial and temporal biases that make it difficult to estimate the transmission dynamics, risk of outbreaks, and geographic burden of disease [65].

Therefore, in order to establish the basis for a holistic approach to the detection of TBDs, improved diagnostics is an important objective. This challenge is strongly reflected in the diverse range of bacteria, viruses, and protozoa responsible for a multitude of acute and chronic pathologies, which in themselves have required a variety of visual, molecular, serological, cell culture, and immunohistochemical tests. This complicates the differential diagnosis of tick exposure and requires the evaluation of multiple types of organisms. In the retrospective analysis of the requests from clinicians from throughout Spain to the National Center for Microbiology (Figure 1), we could observe that in these requests, whose common denominator was tick bites, we found that the majority of requests were for a single pathogenic agent (81.2%; 1111/1368), which was mostly *Borrelia* (46.5%; 517/1111), the causative agent of Lyme Borreliosis; however, a significant proportion of the results of these requests were negative, indicating that there was no broad and targeted diagnosis for the wide range of TBPs.

On the other hand, there is consensus that due to the limitations of existing diagnostics and the increasing threat posed by TBDs, the importance of next-generation technologies in the diagnosis of TBDs is widely recognized [60]. The efficacy of either diagnosis depends largely on the nature of the disease and the methodology used. However, considering the prevalence of co-circulating TBPs, the increasing focus is on reported tick bite exposure and the identification of generic and overlapping symptoms (Figure 3), so there is a marked demand for holistic assays that can detect a wider range of pathogens. In this regard, many molecular diagnostic methods, such as next-generation sequencing, metagenomics, and PCR, promise to improve the detection of new and emerging pathogens with the ability to detect many targets in a single assay. A broad approach to pathogen diagnostic testing can allow the patient sample to be screened for many organisms at once, rather than pooling a variety of tests using multiple methodologies to obtain the same level of diagnostic evaluation. A strategy of catch-all testing also provides an additional level of patient safety, as less tests need to be ordered, and therefore the risk of missing tests or failure to order a particular test is reduced. This can be particularly useful when uncommon clinical presentations or rare etiologies are encountered [65].

The availability of increasingly sophisticated diagnostic tools has transformed our ability to detect and respond to health risks. However, regardless of their sophistication, initial pathogen identification begins in the local community, where human–animal cases occur due to overlapping environments. Unfortunately, many high-risk TBD areas lack the infrastructure and expertise needed to support robust laboratory diagnostic systems, meaning that TBD testing is largely limited to clinical–veterinary laboratories in urban areas, far from the primary interfaces of human–animal–tick interactions. For clinical use, costs and deadlines are the major challenges. Rapid TBD diagnostics are needed to influence the clinical decision process. At present, standard molecular technique timelines are measured in days, limiting their adaptability to acute TBD; in this case, the most appropriate alternative is the use of simultaneous multiple screening using novel molecular techniques that cover the main TBP of the area.

Therefore, it is important to develop multi-focal networks for the surveillance of ticks and TBP and to develop preventive measures, including accurate information on these pathologies as a first course of action.

## 4. Conclusions and Recommendations

Advances in research into new molecular diagnostics will undoubtedly improve our ability to use these powerful analytical tools for clinical care, pathogen discovery, and disease surveillance. The detection of nucleic acids of pathogens using real-time PCR molecular analysis is useful if performed on blood or tissue samples within 5–10 days of symptom onset. In addition, the simultaneous detection of one or more TBP from the same patient sample should become routine when testing for TBDs, especially in specialized centers such as the Spanish National Center for Microbiology, where several TBDs are diagnosed. However, there is a need to improve laboratory capacity, diagnostic tools, and, above all, clinician awareness to detect and control tick-borne diseases from a global health perspective. On the other hand, the harmonization of diagnostic protocols and techniques, together with the establishment of a well-characterized database for the validation of sequence analyses for species differentiation, would contribute to standardizing the analytical process, improving algorithms for both initial diagnosis and final treatment at the individual-patient level.

## Figures and Tables

**Table 1:** Tick-borne diseases based on their etiological agent (bacterium, virus, and parasite) together with the principal tick species that transmits the disease.

	Tick-Borne Diseases		Pathogen	Tick Vector
BACTERIA [11,12,13,14,15,16]	Spotted Fever Group (SFG)	Mediterranean spotted-fever (MSF)	*Rickettsia conorii conorii*	*Rhipicephalus sanguineus s.l.*
		Mediterranean spotted-fever-like	*Rickettsia monacensis* *Rickettsia massiliae* *Rickettsia aeschlimannii*	*Ixodes Ricinus* *Rhipicephalus sanguineus* *Rhipicephalus turanicus* *Rhipicephalus pusillus* *Rhipicephalus bursa*
		Dermacentor-borne necrosis erythema lymphadenopathy (DEBONEL)/tick-borne lymphadenopathy (TIBOLA)/scalp-eschar-and-neck-lymphadenopathy-after-tick-bite (SENLAT)	*Rickettsia slovaca* *Rickettsia raoultii Candidatus Rickettsia rioja*	*Dermacentor marginatus* *Dermacentor reticulatus*
		Lymphangitis-associated rickettsioses (LAR)	*Rickettsia sibirica mongolitimonae*	*Rhipicephalus bursa* *Rhipicephalus pusillus* *H. marginatum*
	Lyme borreliosis (LB)		*Borrelia garinii* *Borrelia afzelii* *Borrelia lusitaneae* *Borrelia valaisaina* *Borrelia burgdorferi s.s*	*Ixodes ricinus*
	Anaplasmosis		*Anaplasma phagocytophilum*	*Ixodes Ricinus* *
	Tularemia		*Francisella tularensis*	*Dermacentor reticulatus* *
	Q fever		*Coxiella burnetii*	*Hyalomma* spp. *
VIRUS [17]	Crimean Congo hemorrhagic fever		Crimean Congo hemorrhagic fever virus (CCHFV)	*Hyalomma* spp. *
PARASITES [18]	Babesiosis		*Babesia microti* *Babesia divergen* *Babesia venatorum*	*Ixodes ricinus*

* These pathogens have been identified in other tick species. For example, Anaplasma phagocytophilum has been detected in Hemaphysalis concinna [19], Francisella tularensis in Ixodes ricinus [20], Coxiella burnetii in Hemaphysalis hispanica, and Rhipicephalus pusillus [21] and CCHFV in Ixodes ricinus and Dermacentor marginatus [22], among others.

## Data Availability

The authors confirm that the data supporting the findings of this study are available within this article.
